# The effect of silver nanoparticles on learning and memory in rodents: "a systematic review"

**DOI:** 10.1186/s12995-023-00381-7

**Published:** 2023-08-01

**Authors:** Farshad Safaei, Javad Farimaneh, Ali Rajabi Mohammad Abad, Ehsan Iranmanesh, Fatemeh Arabpour, Farzad Doostishoar, Zahra Taherizadeh

**Affiliations:** 1 Department of Basic Sciences, Faculty of Veterinary Medicine, Urmia University, Urmia, Iran; 2 Department of Physiology, Faculty of Veterinary Medicine, Shahid Chamran University of Ahvaz, Ahvaz, Iran; 3 The School of Veterinary Medicine, Ferdowsi University of Mashhad, Mashhad, Iran; 4 Neuroscience Research Center, Institute of Neuropharmacology, Kerman University of Medical Sciences, Kerman, Iran; 5 Orthodontics department, school of dentistry, Shahid Sadoughi University of Medical Sciences, Yazd, Iran; 6 Centre of Experimental Medicine, Slovak Academy of Sciences, Bratislava, Slovakia

**Keywords:** Silver Nanoparticles, Learning, Memory, Rodents, MWM, NORT, PAL, Y-Maze, T-Maze, Contextual Fear Conditioning

## Abstract

**Background:**

Silver nanoparticles (AgNPs) are widely used in medicine owing to their antiseptic activity and inducing cell death. Despite AgNPs' importance in nano-engineering and medical benefits, animal studies have shown silver toxicity can damage multiple organs such as the lungs, liver, kidneys, intestines, and brain. Several investigations revealed the correlation between Ag administration by different methods with impaired cognitive and behavioral abilities. Therefore, this systematic review aimed to conclude on the existing evidence of impairments in learning and memory that were changed in rodents exposed to AgNPs.

**Methods:**

Main searches were retrieved in Google Scholar, Scopus, Web of Science, and PubMed databases from 1979 to 2022. Eligibility Criteria were applied to select and extract 15 articles among 892.

**Results:**

Learning and memory abilities of rats and mice in screened studies were evaluated with MWM, NORT, PAL, T-maze, Y-maze, contextual fear conditioning, Radial Arm Maze and Carousel Maze test. Data have shown various sizes from 10 to 100 nm could affect the results of tests among animals exposed to AgNPs compared with control animals. However, in some treatments, results achieved from tests have not demonstrated significant differences between control and treated groups.

**Conclusion:**

Studies have revealed that treatment with Ag-NPs of different sizes can impair learning and memory skills in rats and mice.

## Background

Learning and memory are two of the most essential and important higher brain functions which are closely related together [1]. Learning refers to the process of taking information from the external environment, and on the other, hand memory is the processing of information, storing it, and use of the information later [2, 3]. There are several methods for assessing learning and memory in animal studies (Table 1.) such as shuttle box test for evaluating the passive avoidance learning(PAL), contextual fear conditioning, and Morris Water Maze(MWM) [4]. In the MWM, spatial learning and memory can be evaluated in animals [5]. Radial Arm Maze is another valid test to assess the rodent's memory. Both spatial and non-spatial memory can be evaluated in this task [6]. The Y maze can be used to evaluate short-term memory and spatial working memory in rodents [7]. The other task that is used widely in research studies on rodent’s cognitive ability is the Novel Object Recognition Test(NORT) which is applied to study recognition, learning, and memory, attention, and novelty preferences [8]. Also, for assessing the spatial memory the Carousel Maze is performed and the T-maze task is carried out in rodents to evaluate their spatial working memory [4, 9]. The development of learning and memory skills relies on neurons’ changes either chemically or physically which can be spread through the brain network. This distribution results from potential synaptic connections and neuron communication. So, chemicals and physical alterations play a critical role in the brain’s function and ability such as learning and memory, and these two impaired cognitive skills cause several chronic diseases such as Alzheimer’s disease and Parkinson's disease. Genetic factors that modulate the plasticity of intrinsic excitability likely underlie individual differences in cognitive function and susceptibility to cognitive decline. For example, brain-derived neurotrophic factor (BDNF) stimulates differentiation of neurons, and also enhances learning and memory by adjusting neuronal plasticity. It is clearly stated that genetic features regulate neuroplasticity in the brain and also can make it susceptible to cognitive decline, but intrinsic excitability which modulates learning may be changed by other environmental factors [10, 11].

**Table 1:** Tests and aims

The name of the test	The aim of the evaluation
Morris Water Maze (MWM)	spatial learning and memory
Radial Arm Maze	spatial and non-spatial memory
Y- Maze	short-term memory and spatial working memory
T-Maze	spatial working memory
Carousel Maze	spatial memory
Shuttle Box Test	passive avoidance learning(PAL)
Novel Object Recognition Test(NORT)	recognition, learning and memory
Contextual Fear Conditioning Test	associative fear learning and memory

Nowadays, the wide utility of metallic nanoparticles in biomedical sciences and nanotechnology such as conjugating for antibodies, pharmacological targets, and imaging modalities (MRI, CT, and PET) increase human exposure to heavy metals as toxic and pathologic components. Nano-engineering became common among researchers across the world in recent years owing to rapid technological advances. These kinds of nanoparticles are produced in different sizes that vary from 1 to 100 nm and they can significantly impact on population health [12]. Ag nanoparticles (AgNPs) are applicable nanomaterial in medicine that are used as an antimicrobial production that shows antiseptic activity owing to silver toxicity that induces cell death by making changes in the enzymatic system, and cell permeation. Several studies have shown the toxic effects of AgNP on numerous cell lines such as macrophages, embryonic kidney cells, skin keratinocytes, hepatocytes, neuroblastoma cells, etc. Moreover, animal studies have demonstrated that AgNP administered to animals by different methods such as inhalation, ingestion, or Injections, these nanoparticles detect presence in the blood, and finally lead to toxicity of multiple organs such as the lungs, liver, kidneys, intestines, and brain. In addition, AgNP has high aggregation potential in various organs specifically in brain tissues. The accumulation of AgNPs in the brain is correlated with releasing pro-inflammatory mediators such as Tumour necrosis factor (TNF-α), interleukins and prostaglandins which help AgNPs to pass the BBB (blood brain barrier) easier due to increased permeability of cerebral microvascular. As a result, AgNPs are permitted to transfer through different areas of the brain which would lead to elevated oxidation, apoptosis, and dysregulate the gene expression [13–15]. Meanwhile, silver genotoxicity causes disruptions in the transport of neurotransmitters such as dopamine, norepinephrine, and serotonin in the neural pathway, and any changes in these neurotransmitters may lead to cognitive and behavioral disturbance, especially regarding learning and memory. So, in this study, we reviewed different treatments with AgNPs that may impair learning and memory skills among rodents.

## Methods

### Search strategy

**Fig. 1:**
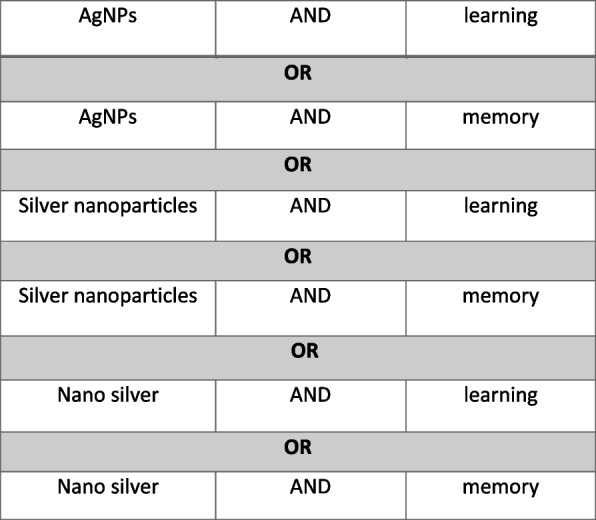
Search terms for article selection

The studies have been systematically searched on online databases in Web of Science, PubMed, Scopus databases, and Google Scholar from 1979 to 2022. All articles were collected with search terms is shown in Fig. 1.

### Inclusion and exclusion criteria

**Fig. 2:**
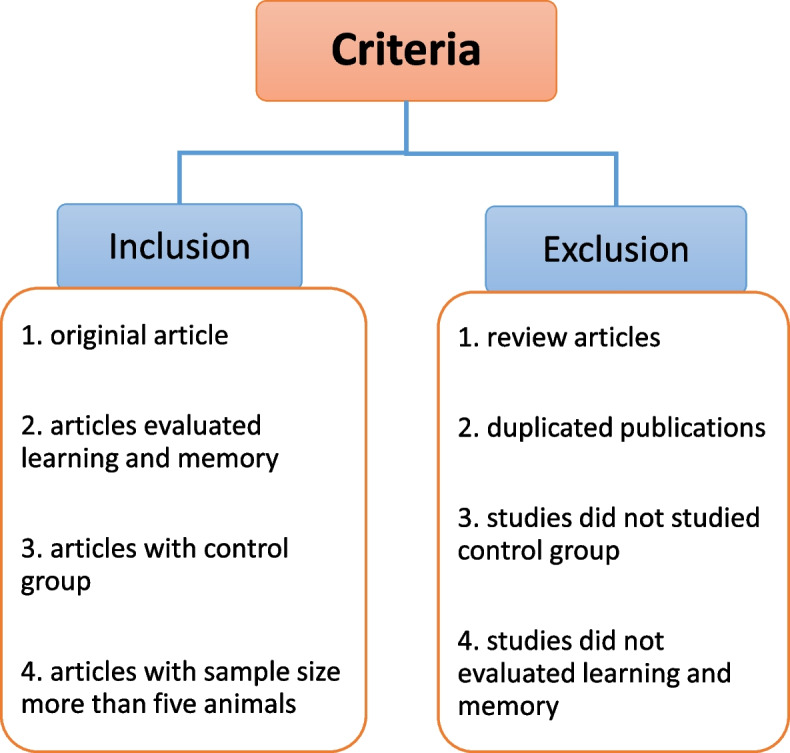
Inclusion and exclusion criteria

Our researchers for retrieving papers were divided into two groups of two members. The first group assessed inclusion and exclusion criteria, and the second group argued on differences that needed to be solved. Finally, those reviewers decided on articles to include or exclude a systematic review study according to criteria. Inclusion criteria: 1. Original articles that evaluate the effect of AgNPs on learning and memory in rodents and published in English 2. No limitation on race and gender 3. Experimental groups should be exposed to AgNPs and the control group did not receive anything, and both of them should be tested for learning and memory. 4 more than five animals were used in each group. Exclusion criteria: 1 Studies do not use a control group. 2 duplicate publications 3. Conference papers 4. Systematic review and meta-analysis. 5 Title and abstract that was irrelevant to AgNPs. 6. data unrelated to learning and memory. All these inclusion and exclusion criteria are shown in Fig. 2.

### Study selection and data extraction

Two reviewers extracted information for each article. The extracted data: 1. basic information for each article including the year of publication, first author's name, animal characteristics (species, gender), the particle size of AgNPs, and methods of learning and memory evaluation. 2. data about treatment such as the method of exposure (injection, inhalation …), treatment duration, and dosage 3. results of learning and memory tests. A third reviewer unraveled contrasts of extracted information. Finally, Characteristics of included studies are showed in Table 2.

**Table 2:** Characteristics of included studies

	**author**	**animal**	**sex**	**age**	**AgNPs size** **(nm)**	**Dose/ days**	**administration**	**test**	**result**
**1**	B. Bouta 2016 [16]	rat	male	adult	10 ± 4	0.2 mg/kg 14 days	gavage	NORT	*p* > *0.05*
**2**	Sh. Khezri 2022 [17]	rat	male	adult	10	50 ppm 100 ppm 200 ppm 400 ppm 21 days	IP	NORT	*p* > *0.05* *p* < *0.05* *p* < *0.001* *p* < *0.001*
								MWM	Significant in sizes > 50 ppm For both learning and memory values
								Y-maze	In treated groups with sizes > 50 ppm: *p* < *0.001*
**3**	M. Ghaleb 2021 [18]	mice	male	adult	19.5 ± 5	40 mg/kg 35 days	IP	T-maze	*p* < *0.001*
								MWM	For both learning and memory values*:* *p* < *0.001*
								PAL	*p* < *0.001*
**4**	K.Dziendzikowska 2021 [19]	rat	male	adult	20 ± 5	0.5 mg/kg 28 days	orally	PAL	*p* < *0.05*
**5**	M. Wesierskaa 2018 [19]	rat	male	adult	20 ± 5	*1 mg/kg **30 mg/kg 28 days	orally	Carousel Maze	*p < *0.001* **p < *0.003*
**6**	L. Davenport 2015 [20]	mice	male	adult	25	50 mg/kg 7 days	IP	NORT	*p* > *0.05*
								MWM	Learning: p > 0.05 Memory: p < 0.05
**7**	L. Hritcu 2011 [21]	rat	male	adult	29 and 23	5 µg/kg 10 µg/kg 7 days	IP	Y-Maze	In both treated groups: p < 0.0001
								Radial arm maze	In both treated groups: *p* < *0.01*
**8**	I. Zinicovscaia 2021 [22]	mice	male	adult	34 ± 1.4	13 mg/ml 4 month 2 month	orally	MWM	Learning: *No difference* Memory: *No difference*
**9**	A. Antsiferova 2018 [23]	mice	male	adult	34 ± 2 and 31 ± 10	2 µg/mL 30 days 60 days 120 days 180 days	orally	contextual fear conditioning	No difference in learning in all groups except 180: *p* < *0.01* No difference in memory in all groups except 180: *p* < *0.05*
**10**	A. Antsiferova 2021 [24]	mice	male	adult	34 ± 5	50 µg/mL 30 days 60 days 120 days 180 days	orally	contextual fear conditioning	Learning in all treated groups: *No difference* Memory: *No difference* *No difference* *No difference* *p* < *0.05*
**11**	P. Liu 2012 [25]	mice	male	adult	36.3 ± 1.2	10 mg/kg 25 mg/kg 50 mg/kg 7 days	IP	MWM	In all groups: Learning: *No difference* Memory: *No difference*
**12**	Y. Liu 2012 [26]	rat	male	adult	32.68 to 38.21	3 mg/kg 30 mg/kg 12 days	Intranasal	MWM	In both treated groups: Learning: *p* < *0.01* memory: *p* < *0.05*
**13**	Kh. Greish 2019 [15]	mice	male	adult	1 to 100 Average:35	mg/kg 2 μg per animal 1 dose 2 doses/ 1 w 3 doses/3 w	IV	MWM	Learning in all treated groups: *p* < *0.0001* *memory:* *p* < *0.05* *p* < *0.005* *p* < *0.001*
**14**	J. Wu 2015 [27]	rat	male	Offspring PND35	20 to 50 Average:35	0.427 mg Ag per g rat During pregnancy	IP	MWM	Learning: *p* < *0.05* memory: *p* < *0.05*
**15**	S. Ghaderi 2015 [28]	mice	Male Female	Offspring PND45-49	5 to 70 Average: 32 ± 6.6	* 0.2 mg/kg **2 mg/kg During Pregnancy (once every 3 days)	Subcutaneous	MWM	Learning: * p > *0.05* **p < *0.01* memory: * p < *0.05* **p < *0.01*
								PAL	*p* > *0.05*

## Result

**Fig. 3:**
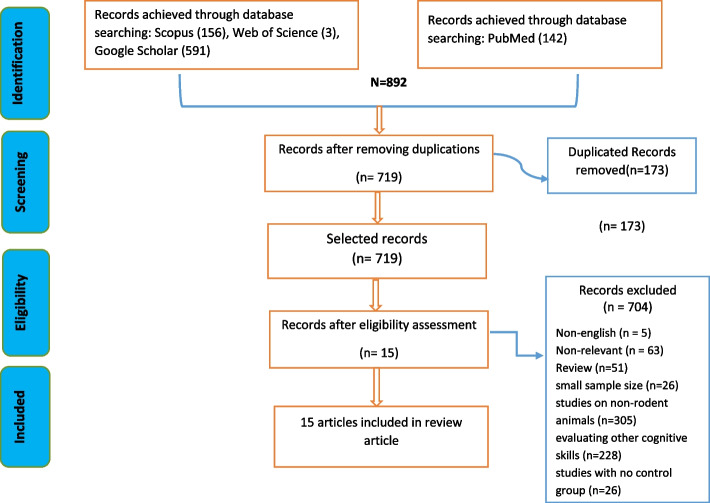
Search strategy

According to the search strategy, 892 articles were recorded from mentioned databases. The title and summary of the articles were considered by reviewers, then, 173 duplicated articles were removed, and 719 articles were considered for further evaluations. After that, the third reviewer excluded 704 documents based on exclusion criteria and 15 articles were included for the systematic review based on this search strategy (Fig. 3.).

### Main outcomes

Beata Dabrowska Bouta in 2016 published data through a study has evaluated recognition learning and memory by a novel object recognition test (NORT) on male Wistar adult rats that received nanosilver with 10 ± 4 nm size and dosage of 0.2 mg/kg by gastric tube for 2 weeks. The administration of saline, Ag + , and nano-Ag have shown all groups were similar in NORT analyzed by one-way ANOVA (*P* > *0.05*). So, it was indicated that a low dose of silver could not induce neurotoxicity and changes in the behavioral evaluation [16].

M. Wesierskaa in the year 2018 revealed a finding from the Carousel Maze test with active place avoidance task was done to assess spatial allothetic memory conducted by cognitive coordination processes among male Wistar adult rats which were treated by AgNPs 20 nm ± 5 nm. According to the method, rats were administered orally in two dosages (1 mg/kg + 30 mg/kg) for 28 days, and data are claimed separately as follows: Rats received 30 mg/kg AgNPs: NOAEL value have shown rats from this group performed significantly lower than control group in long-term memory and short-term memory. Rats received 1 mg/kg AgNPs: Data achieved after administration of 1 mg/kg AgNPs has indicated that administered rats by both 1 and 30 mg/kg AgNPs were impaired in their memory function [9].

Katarzyna Dziendzikowska in 2021 claimed according to the article how spatial memory and learning and memory can be impaired by silver nanoparticles 20 ± 5 nm on male Wistar rats by a dosage 0.5 mg/kg. Assessment of spatial memory with shuttle box test: After oral administration for 28 days, an active allothetic place avoidance task was performed and their findings have demonstrated that indexes have to be decreased showing memory assessment, and through this task, all groups performed significantly different which explained a dysfunctional spatial memory in rats that received AgNPs. As a consequence, impairment in memory in all groups was observed [19].

Lucian Hritcu 2011 has published data about deficits in learning and memory after silver nanoparticles administration intraperitoneal by two different sizes (29 nm, 23 nm) on male Wistar rats with 5 µg/kg and 10 µg/kg (both dosages for both sizes) during a week. After that, Y-maze Task and radial arm-maze task were used to evaluate impairments. As a result, through the Y-maze trial, recorded information was reported a remarkable alternation of spatial memory function among rats treated with 5 and 10 doses and both sizes. So, that has been understood by a significant reduction in the continuous change percentage compared with rats in the control group has indicated how short-term memory was impaired. Although groups administered by silver have shown noticeable changes in comparison with control ones, both silver receiver groups were similar in result. Through the radial arm-maze trial, working memory errors (WME) have illustrated a significant different interaction between groups that revealed short-term memory deficient. Meanwhile, both groups treated with AgNPs demonstrated more WME than the control group and they had no significant differences in comparison with each other [21].

Laurie L. Davenport in the year 2015 designed a study on male C57BL/6 mice by Ag intranasal administration. So, particles were used with the size of 25 nm, and a dose of 50 mg/kg was applied by both single doses and repeated doses for 7 days. Finally, spatial learning and memory were assessed by NOR and MWM tests. Findings based on NORT have revealed no significant differences in preference for the novel object in rats who received 50 mg/kg AgNPs for a week compared with the control group. Also, according to data after MWM, there were no remarkable differences among each group on day 1, and travel distances among them were not significantly different which suggested no disability in spatial learning had happened. After the learning phase, a probe trial was performed to evaluate spatial memory that indicated rats treated with 50 mg/kg AgNPs spent less time significantly in the target quadrant. As a result, the probe trial has displayed a reduction in spatial memory through silver toxicity [20].

Mohsen Ghaleb in the year 2021 performed a research project on male mice which were intraperitoneally injected for 35 days with Ag-NPs (40 mg/kg) (19.5 ± 5 nm). After day 35, behavioral evaluation was carried out to assess neurotoxicity induced in mice. As a result, mice exposed to Ag-NPs have shown weak performance through memory tasks in the T-maze experiment. All value parameters including the number of entries into the main arm and food arm, additionally the time taken to spend time in the food arm were significantly different among mice with Ag-NPs administration compared to control mice. Data achieved from shuttle box test have demonstrated that the number of intertribal, stimulated, and reinforced crossings were noticeably decreased in mice treated by Ag-NPs than control group. Also, the males exposed to silver nanoparticles have shown poor learning ability due to spending more time responding to the shock in comparison to the control group. The third evaluation was done by MWM. Collected data from this test have revealed longer escape latencies among longer escape latencies which means impaired learning skills. Also, the probe test time was recorded longer in the treated group compared to the control group, and that displayed an impairment in spatial memory in mice. In conclusion, all these three behavioral tests clarified cognitive behaviors were altered through Ag-NPs [4].

In the year 2022, Shiva Khezri was successful to evaluate silver nanoparticle toxicity on male Wistar rats which were intraperitoneally exposed to Ag-NPs(10 nm) for 21 days with three different dosages of 50, 100, 200, and 400 ppm. The released data from NOR test and Y-maze task have demonstrated that all three administered dosages of Ag-NPs (100, 200, and 400 ppm) were significantly effective in decreasing recognition learning and memory tasks and spatial working memory. Additionally, MWM tests have revealed groups exposed to Ag-NPs with the dosage more than 50 ppm performed with impaired learning skills compared to their control rats during tests based on value parameters including the traveled distance, and escape latency of the training days. Likewise, the time spent in the target quadrant, which give data about spatial memory, shown a noticeable reduction among rats treated with AgNPs (the dosage more than 50 ppm). Although both values were significantly increased among treated rats, the swimming speeds had shown no noticeable differences. So, data obviously displays how Ag-NPs can adversely affect all recognition and spatial learning and memory among male rats treated with dosages more than 50 ppm [17].

In 2012 Ye Liu explained how a low dose (3 mg/kg) and a high dose (30 mg/kg) of silver nanoparticles can cause detrimental impacts on spatial learning and memory. Through this study, male Wistar adult rats received Ag-NPs with the sizes from 32.68 to 38.21 nm and two different dosages for 12 days by nasal administration. Then, the MWM task was applied to evaluate silver neurobehavioral toxicity. Based on the escape latency measurement, all three groups passed this index progressively from day 1 to 4. While both low dose and high dose of silver influenced spatial learning detrimentally in comparison with the control group, control rats could record lower escape latencies. Also, there was a difference among rats who received 3 and 30 mg/kg that is not significant and low doses had been reported with lower records in the same measurement. After the spatial learning phase, a probe test was done to assess the spatial memory that revealed there were two significant differences in both time percentages spent in the target quadrant and the number of crossings on the hidden platform in rats treated by Ag than control ones. In other words, both measurements have decreased among rats who received silver nanoparticles in comparison with the control group, also a high dose of silver was obviously lower. In conclusion, both phases in spatial cognition evaluation could indicate how Ag-np exacerbates spatial skills with various dosages [26].

According to recent research done by Anna A. Antsiferova in 2021, silver nanoparticles with a size of about 34 ± 5 nm can influence C57BL adult male mice's cognitive ability. So, it was evaluated by contextual fear conditioning at a dose of 50 µg per day. There were four experimental groups as follows: rats treated orally by Ag-NP for 30, 60, 120, and 180 days. Consequently, these findings have proven learning ability was not changed by Ag-NPs. Nevertheless, according to data from the long-term contextual memory, there were no remarkable differences in control rats in comparison with Ag-NP groups at 30 days, exceptionally, a significant difference was reported at 180 days of administration which had indicated impaired memory through silver toxicity [24].

Anna Antsiferova during the year 2018 conducted an exploration including four different groups of C57Bl/6 eight-week-old male mice which were orally treated with silver nanoparticles every day with the size 34 ± 2 nm and at concentrations of 2 µg/mL. These mice were divided into four groups which had received Ag-NPs respectively for 30, 60, 120, and 180 days. At the end of each period, the Light–Dark Box test was done to assess learning and memory parameters. According to the similar information received from 30, 60, and 120-days groups, the number of freezing acts was low before performing the electric pulse among both control and treated mice, after the electric pulse, the number of freezing acts significantly increased in both groups which mean Ag exposure for 30 days did not affect the quality of learning in male mice. Also, a slight decrease was observed in both groups in the number of freezing acts in learning during the 24 h time-course. Therefore, contextual memory was not impaired. However, based on data from mice exposed to silver nanoparticles for 180 days, it was concluded that both contextual learning and memory were noticeably impaired [23].

Peidang Liu in the year 2012 by MWM task on adult male ICR mice could evaluate silver detrimental effects in spatial learning and memory, while the particles size was 36.3 ± 1.2 nm, and dosage was determined for three different groups as follows: 10,25, and 50 mg/kg Intraperitoneal administered for 7 days. There was a significant difference between group and day, however, there were no significant differences among mice that experienced Ag exposure, therefore, spatial cognition with no impairment performed perfectly [25].

Jinjin Wu in 2015 started to appraise how silver toxicity during pregnancy can affect male offspring's spatial learning and memory assessed by MWM. So, Ag-NPs were made in the size of 20–50 nm, and have been intraperitoneal administered to mothers in four forms: polyvinyl pyrrolidone (PVP)-coated, uncoated-NPs (0.427mgA pregnant), Ag NPs (0.407 mg/g rat), silver nitrate (0.013 mg/ g rat). Then, male offspring at postnatalday35(PND35) were entered into MWM, and two-way ANOVA analyzed data from the average escape latency to indicate any disability in spatial learning. While data after the third test day has shown a significant increase in the escape latency among rats who received uncoated Ag-NPs compared with other rats treated with a silver. As a result, uncoated Ag-NPs were found as a detrimental factor in the spatial learning of rat offspring. Similarly, in the probe trial rats administered with the uncoated Ag-NPs revealed a significant reduction in spent time in the target quadrant than all other groups, and other groups have shown no reliable difference. In sum, hippocampal neurodevelopment and spatial ability related to cognition can be damaged in rat offspring, if maternal exposure to Ag- NPs during pregnancy has occurred [27].

Similar to Jinjin Wu’s study, Ghaderi in the year 2015 studied NMRI mice exposed prenatally to high doses of silver nanoparticles (0.2 and 2 mg/kg of body weight (BW)) and subcutaneous injection with the size between 5–70 nm. So, female mice were receiving Ag-NPs from gestation day 3, and it was repeated once every three days until parturition. When offspring from both male and female gender were 45 to 49 days old, MWM and passive avoidance learning (PAL) tests were applied to assess their spatial cognition. According to statistical data from the MWM task during 4 days, performances during training trials were improved by a decreasing trend among all rats, although this trend was significantly different. Also, the average moved distance was different in rats that received 2 mg/kg Ag-NPs compared with the control group. The swim average speed during continuous training trials, while there was no statistical variation among rats. Additionally, the Ag-NPs administration was not dependent on sex at during the task. Consequently, mice performances were not affected by gender, and showed a valuable difference for the percentage of traveled distance in the target quadrant). Both distance and the time spent in the target quadrant were recorded lower significantly in rats treated with 2 mg/kg Ag-NPs compared with the control group. A remarkable difference was reported between male and female offspring receiving silver. In conclusion, not only Ag-NPs impaired spatial learning, but also it can detrimentally change spatial memory. Based on the second behavioral task, PAL data analyzed by two-way ANOVA (treatment × sex) has demonstrated that Ag-NPs have not changed indexes evaluated in passive avoidance learning [28].

In 2019 Khaled Greish used Adult male BALB/C mice to evaluate the neurotoxicity of silver nanoparticles. So, they designed this study to administer Ag-NPs in various sizes from 30 to 40 and at a very low dosage (0.1 mg/kg, 2 μg per animal) intravenously. Also, the administration has been conducted by a single injection, 2 injections with a week interval, and 3 injections over 3 weeks. Consequently, rats in the control group were observed normally during the learning assessment and could pass the latency with a reduced trend to find hidden the platform in the pool between the first trial. However, rats that received Ag-NPs were not successful to pass this time with a decreased shift from the initial to the end. Similarly, the distance traveled by rats to reach the submerged platform was recorded to obey a decreased trend, and the AgNPs treated rats could not follow this normal pattern. With a comparison in the escape latency between control rats, and rats receiving one AgNPs administration. But this measurement has not shown any significant variation between the groups treated with one or two AgNPs injections, or between the ones treated with two or three AgNPs administrations. Also, the swimming speed measurement was not observed significantly different among all rats, which indicated that the lower swimming speed has not played an effective role to prevent a reduction trend in the escape latency, and abnormal data from time is associated with learning disability. Owing to the probe trial data, after eliminating the submerged platform, the total time spent was recorded in all 4 quadrants, and time spent by rats in the target quadrant, where the platform was located, could suggest how spatial memory was altered abnormally. In conclusion, analyzed outcomes from this behavioral test directed our finding to impaired spatial learning and memory induced by silver nanoparticles [15].

In 2021, Zinicovscaia managed a project on mice to evaluate the chronic effects of Silver Nanoparticles on cognitive skills. Those mice were exposed to Ag-NPs with a specific concentration (13 mg/ml) and size of 34 ± 1.4 nm by oral Administration through drinking water. One group was treated with silver nanoparticles for 2 months, and the second group for 4 months. At the end of the treatment, a MWM test was applied on mice for 3 groups. Results from the MWM of selective tests have shown that the total moved distance to the platform and the distance to the platform in both groups treated by silver nanoparticles was significantly different compared to the control groups. Then, through evaluating the latency to the platform, it was revealed that both the 2-month experiment and the 4-months experiment revealed significant differences than control mice. Although, other parameters’ values were not significantly noticeable from each day. Results from the MWM of the main test have statistically demonstrated no significant differences in parameters’ values among experimental groups compared to controls. So, it is concluded that Ag-NPs with a dosage of 13 mg/ml and size of 34 ± 1.4 nm in mice treated for 2 and 4 months do not affect spatial learning and memory [22].

## Discussion

After 47 different kinds of administration, 8 tasks evaluated learning and memory (Table 3.). According to the achieved data, 30 treatments with AgNPs indicated a significant alteration in those cognitive skills, while 17 treatments did not illustrate significant impairment among rats. So, we categorized all factors that are effective in AgNPs neurotoxicity induction among rodents and explained them based on variations in sizes and doses of AgNPs, exposure duration to AgNPs, sex, species and strains of animals. Then, immunological mechanism and pathways which contributed in AgNPs toxicity were discussed in addition to changes occurred and assessed by genomics.

**Table 3:** Tasks and results

	***Significant***								***Non-significant***			
**MWM** Different treated groups: 19 Sig:13 Non-sig:6	100 ppm		21 days			10 nm		Learning memory	50 ppm	21 days	10 nm	Learning memory
	200 ppm		21 days			10 nm		Learning memory	50 mg/kg	7 days	36.3 ± 1.2 nm	Learning memory
	400 ppm		21 days			10 nm		Learning memory	13 mg/ml	4 month	34 ± 1.4 nm	Learning memory
	40 mg/kg		35 days			19.5 ± 5 nm		Learning memory	13 mg/ml	2 month	34 ± 1.4 nm	Learning memory
	50 mg/kg		7 days			25 nm		memory	10 mg/kg	7 days	36.3 ± 1.2 nm	Learning memory
	3 mg/kg		12 days			32.68 to 38.21 nm		Learning memory	25 mg/kg	7 days	36.3 ± 1.2 nm	Learning memory
	30 mg/kg		12 days			32.68 to 38.21 nm		Learning memory				
	mg/kg 2 μg per animal		1 dose			35 nm		Learning memory				
	0.1 mg/kg 2 μg per animal		2 doses/ 1 w			35 nm		Learning memory				
	0.1 mg/kg 2 μg per animal		3 doses/3 w			35 nm		Learning memory				
	0.427 mg Ag per g rat		During pregnancy			35 nm		Learning memory				
	2 mg/kg		During pregnancy (once every 3 days)			32 ± 6.6 nm		Learning memory				
	0.2 mg/kg			During pregnancy (once every 3 days)		32 ± 6.6 nm		memory				
**NORT** Different treated groups :6 Sig:3 Non-sig:3	100 ppm			21 days		10 nm		Learning memory	0.2 mg/kg	14 days	10 ± 4 nm	Learning memory
	200 ppm			21 days		10 nm		Learning memory	50 ppm	21 days	10 nm	Learning memory
	400 ppm			21 days		10 nm		Learning memory	50 mg/kg	7 days	25 nm	Learning memory
PAL Different treated groups: 3 Sig:2 Non-sig:1	40 mg/kg			35 days		19.5 ± 5 nm		Learning memory	0.2 mg/kg	During pregnancy (once every 3 days)	32 ± 6.6 nm	Learning
	0.5 mg/kg			28 days		20 ± 5 nm		Learning memory				
Contextual Fear Conditioning Different treated groups: 8 Sig:2 Non-sig:6	2 µg/mL			180 days		34 ± 2 and 31 ± 10 nm		Learning memory	2 µg/mL	30 days	34 ± 2 and 31 ± 10 nm	Learning memory
	5 µg/mL			180 days		34 ± 5 nm		memory	2 µg/mL	60 days	34 ± 2 and 31 ± 10 nm	Learning memory
									2 µg/mL	120 days	34 ± 2 and 31 ± 10 nm	Learning memory
									5 µg/mL	30 days	34 ± 5 nm	Learning memory
									5 µg/mL	60 days	34 ± 5 nm	Learning memory
									5 µg/mL	120 days	34 ± 5 nm	Learning memory
Y-Maze Different treated groups: 6 Sig:5 Non-sig:1	100 ppm	21 days			10 nm		memory		50 ppm	21 days	10 nm	memory
	200 ppm	21 days			10 nm		memory					
	400 ppm	21 days			10 nm		memory					
	5 µg/kg	7 days			29 and 23 nm		memory					
	10 µg/kg	7 days			29 and 23 nm		memory					
**T-Maze** Different treated groups: 1 Sig:1 Non-sig:0	40 mg/kg	35 days			19.5 ± 5 nm		memory					
**Carousel Maze** Different treated groups: 2 Sig:2 Non-sig:0	1 mg/kg	28 days			20 ± 5 nm		memory					
	30 mg/kg	28 days			20 ± 5 nm		memory					
**Radial Arm Maze** Different treated groups:2 Sig:2 Non-sig:0	5 µg/kg	7 days			29 and 23 nm		memory					
	10 µg/kg	7 days			29 and 23 nm		memory					

### Influence of the size

A study discussed the various sizes of AgNPs including a wide range from small size of 10 nm to the large size of 50 nm. Their findings indicate that silver is affected by the different bio-relevant conditions. Nanoparticle aggregation has a huge influence on their biological activity. Remarkably, the larger particles demonstrated a higher resistance to external influences. Larger particles have a more pronounced impact than smaller ones. Studies have been conducted in vitro on cytotoxicity and antibacterials. These studies were done by treating cells with nanomaterial accumulation at different stages of development. Thus, the toxicity of AgNPs results in a complete loss of biological activity and higher levels of aggregation resistance observed for larger particulates have had a significant influence on in vitro toxicity, because these samples retain more activity against bacteria and mammalian cells. These results show that, despite the prevailing opinion of the literature on nanomaterials, it is not feasible to aim for the smallest possible particle size. The increase in size of nanomaterials can significantly reduce the rate of aggregation, which is inevitable within biological systems and natural environments. The present common approach in the relevant literature, which promotes the concept of 'less is more' with regard to biological uses of nanomaterials, calls into question this conclusion. Although it is well known that smaller nanoparticles of the same bioactive chemical composition have a higher toxicity, results suggest that there is a significant difference between toxicity and longevity if sustained biological effects are desired. This suggests that the balance needs to be achieved with this trade off, which would have a major impact on this view of nanoparticle toxicity in scientific research [29]. Moreover, another study explored that AgNPs induced cytotoxicity in the culture of endothelial cells in the rat brain and the problem arises from citrate coated spherical AgNPs. They cause cell damage to the membrane and affect colony formation of RBE4. In assessing membrane damage by Neutral Red (NR) uptake assays, it was shown that a significant decrease in dye uptake had been achieved when exposed to Ag10 compared with untreated cells. The effect has been found to be dependent on particle size, particle surface area, dose and exposure time. Likewise, the dose dependent decrease of NR uptake when exposed to AgNPs (7–10 nm) was observed in use of HepG2 (a human liver derived cell line) [30].

### Influence of the dose

As mentioned in the previous paragraph, various doses of the AgNPs induce different effects and there are more studies about biological toxicity of green synthesized silver nanoparticles in rat’s brains which varies depending on doses. For example, an examination of the toxicity of green synthetic AgNPs on cortical and hippocampal levels of oxidative stress markers and the activity of acetylcholinesterase (AchE) and monoamine neurotransmitters (DA, NE, and 5H-T) have been done. Then, AgNPs showed minimal oxidative stress in the cortex and hippocampus at the doses administered. However, AgNP showed a dose dependent inhibitory effect on the activity of the AchE and a decrease in 5Ht and NE levels by administration of the low (0.5 mg/kg), the medium (5 mg/kg) and the high doses (10 mg/kg). Therefore, the rate of AgNP synthesis was determined to be associated with dose when examining brain tissue. However, Nitric oxide increased in the cortex only when rats were treated with high doses of AgNP(10 mg/kg), and a significant change was not observed at those two doses (0.5 mg/kg and 5 mg/kg). Therefore, 5H-T and norepinephrine have been significantly reduced at the high dose of 10 mg kg. It is known that alterations in monoamine neurotransmitters may be involved in many neurological disorders [31]. More exploration on rat's brains found AgNPs impaired mitochondrial function through autophagy even with a low dose (0.2 mg/kg). Accordingly, the autophagy was confirmed by protein markers including: beclin 1 and microtubule-associated proteins 1A/1B light chain 3B which were expressed more than a normal level. In addition to molecular findings, pathological investigations showed changes in cellular morphology such as swelling that results in reduced ATP and energy [32]. There is a study that assessed hematological and biochemical factors in rats exposed to two doses of AgNPs, then evaluated histopathological changes in their brain tissue. So, data revealed that both low (100 mg/kg/day) and high (500 mg/kg/day) doses of AgNPs accumulated in the brain and induced significant cellular alterations [33].

### Influence of the exposure duration

According to several studies, the time of AgNPs administration is directly correlated with the toxicity and brain dysfunction. There is some information which is provided by a research study about neuronal changes in rat’s brains with diabetic who were exposed to AgNPs with a dose of 10 mg/kg everydays for 1 month [34]. Previous studies have demonstrated that chronic intranasal administration of a daily dose of 1 mg/ kg of AgNPs for 12 weeks results in silver accumulation in the Sprague–Dawley rat’s brain for a long time. Nasal exposure from olfactory epithelium is the main method for inducing neurotoxicity and AgNPs can distribute into the brain tissue with a high concentration after a chronic administration [35]. Similarly, a study on Sprague–Dawley rats exposed to three different dosages of AgNPs showed inhalation toxicity and a high concentration of silver in the brain within 28 days in a group that received high doses of AgNPs [36]. Also, another exploration revealed that the daily gastric administration of AgNPs with a dose of 0.2 mg/kg after 14 days was accumulated in hippocampal and cerebral regions among rats [37].

### Influence of the gender

There are few studies that assess the cognitive and behavioral impairments among female animals exposed to AgNPs and researchers ordinarily prefer male animals. According to the results and other previous articles discussed above in different sections, male rats that have been administered by different doses of AgNPs, especially during a long term, have shown significant changes in their brain tissue and their behavior. Although, we found two articles which compare data from male and female animals in order to conclude about sex differentiation and impaired learning and memory with AgNPs exposure that observed no significant differences between male and female [36, 38]. Moreover, there are a couple of limited articles on female animals which received AgNPs during their pregnancy and then offspring were evaluated by PAL and MWM and showed impaired learning and memory abilities [28].

### Influence of the species and strain

The main aim in this systematic review was the learning and memory assessment of rodents exposed to AgNPs. As we evaluated selected articles two different species including rats and mice were used in those articles and that were also different in their strain.

### Rats

The result from an exploration demonstrated that exposure to AgNPs and ZnONPs among male albino rats caused a remarkable change in their stress oxidative assessments in their brain [34]. Also, sprague–dawley rats were used in several research projects for assessment of AgNPs neurotoxicity and histopathological examinations depicted AgNPs were increased in the sprague–dawley rats' brains in both genders [35, 36].

### Mice

Furthermore, mice were suitable animals for evaluation of AgNPs toxicity in many organs such as the brain. As published data explained that AgNPs induced some changes in the neuronal system of Balb/C mice through making the blood brain barrier (BBB) more permeable. After the injection, histopathological finding from brain tissue showed astrocytes were changed with a noticeable edema in those regions [39]. Additionally, more evidence presented the AgNPs had no effect on neurons among C57BL/6 J mice by increasing ROS and inflammatory factors or decreasing the BBB integrity. Nevertheless, behavioral assessment revealed no significant impairments in open field task and the X-maze task, except motor function test [38].

### Genomics

According to the previous explanations, the performance of neural cells in the brain was changed during exposure to AgNPs. The cell’s function is directly associated with gene expression and related products which regulate the cell’s role, so any alterations and abnormal output remind a change in the genome. For this reason, transcriptomic gene-network data potentially clarify these huge changes occurred into cells exposed to AgNPs.

There is some information from gene-network analysis which are provided by several studies about neurodegeneration in neurons of mouse received AgNPs. As a result, the expression of the gene of interferon regulatory factor 1 (IRF1) as an important immunological factor in AgNPs neurotoxicity was altered in three kinds of neural cells. Likewise, the data collected from real-time PCR revealed that the gene expression of RasGRF1 was noticeably increased after AgNPs administration and BCL2 genes were expressed significantly fewer. Furthermore, western blot results demonstrated that amyloid precursor protein (APP) was induced in cells exposed to AgNPs [40]. Additionally, AgNPs exposure affects DNA methylation and the gene expression of histone via data obtained from genome-scale assessment in mice [41]. There is some information which is provided by a research study on human cells to assess the AgNPs toxicity and immune response with mass cytometry and scRNA-seq. Consequently, the transcriptomic data showed genes which contributed with metabolism, apoptosis and oxidative stress were changed in their expression after receiving AgNPs and a relation was reported between AgNPs dose and cell responses to the toxicity [42]. A similar result was obtained in the study on mice that reported the gene expression of oxidative stress factors such as NADPH oxidase, glutathione reductase and glutathione peroxidase were increased after exposure to AgNPs [43]. As published data explained that AgNPs neurotoxicity among mice is in accordance with the PI3K/AKT/mTOR signaling pathway in their hippocampus and also the autophagy which was started after phosphorylation and increasing in caspase-3 and Bax alongside a decreasing in Bcl-2. This information was collected by western blot and RT-qPCR evaluations that showed the mRNA and proteins level of p-PI3K p85 (Tyr458), p-AKT (Ser473), p-mTOR (Ser 2448) were higher, while the expression of PI3K p85, AKT, mTOR was lower in the AgNPs group than the control group. All these changes resulted in the activation of the PI3K/AKT/mTOR signaling pathway. Moreover, the phosphorylation occurred more about PI3K, AKT and mTOR while gene expression of PI3K, AKT and mTOR was observed less in mice receiving the AgNPs. Besides, autophagy was detected by both TEM and the gene expression evaluation. An increased level of LC3-II/I and a reduction in p62 and Beclin-1 expression in AgNPs group endorsed that the autophagy was induced [44, 45]. Another pathway which is the correspondence of AgNPs neurotoxicity called endoplasmic reticulum (ER) stress which is tracked by a PCR array and the gene expression and western blotting on mice. The data from these tests revealed that CHOP/DDIT3 gene expression, eIF2a, Caspase-12 and CHOP proteins were activated at the same time after exposure to AgNPs [46]. Regarding an article discussed the neuronal differentiation of SH-SY5Y cells and kinase signaling pathways, the treatment of these cells with AgNPs caused a significant enhancement of Map-2, β-tubulin III, synaptophysin, neurogenin-1, Gap-43, and Drd-2 gene expression that are important markers of neuronal differentiation [47].

### Immunology and pathways

Many studies on AgNP have indicated that it had induced neurotoxicity in both animal experiments and in vitro models by increasing oxidative stress and production of radical oxygen species (ROS) in the neurons, especially when animals were exposed to high doses. Then, AgNPs were found in an accumulated form in the neuronal cells of rats and their brain’s endothelial cells. More exploration in the brain tissue of poisonous rats have revealed that lamellae of myelin sheaths were contaminated by AgNP as well. On the other hand, the stabilization of myelin structure is dependent on some kinds of protein and any changes in these proteins can result in structural disruption in myelin sheaths and makes it vulnerable to ROS which has been shown in AgNP toxicity. The more ROS is produced, the more antioxidant defense systems have to work against the oxidation process, and an excessive ROS activity by AgNP causes an imbalanced condition and modifies DNA, proteins, lipids, and other vital molecules which finally demonstrate a dysfunction among cells. According to the data collected through assessment of biochemical factors in plasma, MDA level was significantly increased due to changes in the peroxidation and the damage to lipids of myelin membranes in neural cells exposed to AgNP. Moreover, a decrease was investigated in protein-bound –SH groups, whether because of interactions between protein groups and AgNPs, or due to the oxidative stress. Also, sulfhydryl groups provide the proteins with a situation for elevating metal binding probability [48]. Other studies based on molecular assessment have reported that the administration of AgNPs in long-term can significantly change the levels of neurotransmitters including dopamine, serotonin, and acetylcholine, and a reverse correlation was observed between two of them, a high level of dopamine was associated with a low amount of serotonin. Clearly, dysregulation of serotonin and dopamine is directly connected with clinical symptoms in humans such as depression, anxiety, and learning impairments. More evaluation of immunological factors through serology assessment have described the association between pro- and anti-inflammatory cytokines in plasma and the disruption of the integrity of the BBB in animals exposed to AgNPs. In other words, a high concentration of pro-inflammatory cytokines (IL-1β, IL-5, IL-6, IL-12(p70), TNF-α, GM-CSF, and G-CSF) have shown that immune system cells such as macrophages, and B cells were activated more than the normal level and these inflammation and cell’s activities caused a perforation in cells and decreasing the junction. Consequently, dysfunction of neurons is revealed with impaired memory and brain function [20]. It has been studied that synaptic plasticity of the hippocampus directly regulates spatial learning and memory. As neurobehavioral studies have shown the mechanism of learning and memory, hippocampus has the main responsibility of formation and maintenance of spatial data, so, degeneration in hippocampus plasticity can significantly decline spatial cognition. On the other hand, spatial learning and memory are controlled by dopamine in the rodent's hippocampus and rewards and punishment conduct the learning process. The activation of N-methyl-d- aspartate (NMDA) receptors have caused changes in intracellular calcium signaling pathways due to AgNPs toxicity in rats receiving orally AgNPs. Besides, cell dysfunction and brain damage lead to the death of cells in main regions, particularly in the hippocampus where the differentiation of neurons and producing dopamine was reported significantly altered. After that, dysfunctional conditions of mitochondria, increased apoptosis, and hyperpolarization after alternation in voltage-gated sodium channels have been revealed and dysfunction in motor activity, spatial memory and passive avoidance learning was observed in rats exposed to AgNPs [9, 28]. According to the pathological findings from hippocampal neurons, several changes including a wide edema, shrinkage in nucleus, and necrobiosis were observed after AgNPs administration. More data has suggested that ATP synthesis clearly was disrupted after the changes in the mitochondrial respiratory chain and caused DNA damage. Also, jun-N terminal kinase (JNK) pathway which is dependent on mitochondria's activity causes apoptosis with AgNPs toxicity, and the connection among a huge population of neurons has remarkably weakened that negatively affects spatial cognition ability [26]. Molecular and genetics study successfully assess the level of the hippocampal GAP-43 mRNA and protein expression among rats exposed to AgNPs which made a hindering in synaptic plasticity and then cognitive decline because neurons were disable in regeneration and developing of their connection and synapse [27]. There is some evidence that showed Ca2 + activity in neurons trigger the GSK-3β phosphorylation which also was increased via oxidative stress productions such as NOS and the PI3-kinase pathway down regulation that were induced via AgNPs and helps biomarker assessment to easily follow up AgNPs toxicity. Then, other useful data has been achieved about Tau hyperphosphorylation which declined axonal transport and microtubule stability that were directly associated with cognition deficient. All these pathological changes result from AgNPs exposure such as what happens in neurodegenerative disease [49]. In other studies, that AgNPs intoxicated rats were orally exposed and increased Glu and Asp as excitatory amino acids neurotransmitters, and reduced GABA and Gly as inhibitors in neuro-transportation in them, at the end, these molecular changes had revealed severe damage to the brain and cognitive abilities [50]. According to previous exploration, oxidative damage remarkably induced DNA damage and extracellular signal-regulated kinase (ERK) was known as a pathway which improved DNA repair. Likewise, silver nanoparticles were studies through many cellular investigations which explained MAPKs activity and p-p38 MAPK protein expression were significantly increased in T-cells exposed to AgNPs which is linked with Cytokines, Interleukins and TNF-α [51, 52].

## Conclusion

This systematic review indicated an outline of accessible investigations performed on learning and memory impairments induced by silver nanoparticles on rodents. Available studies have shown rats, treated with Ag-NPs with a diameter in the range from 1 to 100 nm, behaved different significantly through tasks including MWM, NORT, PAL, T-maze, Y-maze, radial arm maze, Carousel Maze, and contextual fear conditioning that evaluated their learning and memory. These impairments are related to changes in gene expression, signaling pathways, inflammation and oxidative stress. Additionally, variation in some other factors such as sizes and doses of AgNPs, exposure duration to AgNPs, sex, species and strains of animals can result in various levels of toxicity. To confirm this conclusion, a meta-analysis study is suggested in order to investigate the learning and memory ability among rodents exposed to AgNPs.

## Data Availability

The materials during this review study are available from the corresponding author on reasonable request.

## Abbreviations

PAL: Passive Avoidance Learning
MWM: Morris Water Maze
NORT: Novel Object Recognition Test
BDNF: Brain-Derived Neurotrophic Factor
AgNPs: Ag Nano Particles
TNF-α: Tumor Necrosis Factor-α
BBB: Blood Brain Barrier
ROS: Radical Oxygen Species
NMDA: N-Methyl-D- Aspartate
JNK: Jun-N Terminal Kinase
ERK: Extracellular Signal-Regulated Kinase
